# Inhibition of AKT Signaling Alters βIV Spectrin Distribution at the AIS and Increases Neuronal Excitability

**DOI:** 10.3389/fnmol.2021.643860

**Published:** 2021-06-30

**Authors:** Jessica Di Re, Wei-Chun J. Hsu, Cihan B. Kayasandik, Nickolas Fularczyk, T. F. James, Miroslav N. Nenov, Pooran Negi, Mate Marosi, Federico Scala, Saurabh Prasad, Demetrio Labate, Fernanda Laezza

**Affiliations:** ^1^Department of Pharmacology and Toxicology, University of Texas Medical Branch, Galveston, TX, United States; ^2^Biochemistry and Molecular Biology Graduate Program, Graduate School of Biomedical Sciences, University of Texas Medical Branch, Galveston, TX, United States; ^3^M.D./Ph.D. Combined Degree Program, Graduate School of Biomedical Sciences, University of Texas Medical Branch, Galveston, TX, United States; ^4^Department of Mathematics, University of Houston, Houston, TX, United States; ^5^Department of Computer Engineering, Istanbul Medipol University, Istanbul, Turkey; ^6^Department of Electrical and Computer Engineering, University of Houston, Houston, TX, United States

**Keywords:** AIS plasticity, support vector machine classification, confocal imaging, GSK3 – glycogen synthase kinase 3, WEE1 kinase, patch clamp electrophysiology

## Abstract

The axon initial segment (AIS) is a highly regulated subcellular domain required for neuronal firing. Changes in the AIS protein composition and distribution are a form of structural plasticity, which powerfully regulates neuronal activity and may underlie several neuropsychiatric and neurodegenerative disorders. Despite its physiological and pathophysiological relevance, the signaling pathways mediating AIS protein distribution are still poorly studied. Here, we used confocal imaging and whole-cell patch clamp electrophysiology in primary hippocampal neurons to study how AIS protein composition and neuronal firing varied in response to selected kinase inhibitors targeting the AKT/GSK3 pathway, which has previously been shown to phosphorylate AIS proteins. Image-based features representing the cellular pattern distribution of the voltage-gated Na+ (Nav) channel, ankyrin G, βIV spectrin, and the cell-adhesion molecule neurofascin were analyzed, revealing βIV spectrin as the most sensitive AIS protein to AKT/GSK3 pathway inhibition. Within this pathway, inhibition of AKT by triciribine has the greatest effect on βIV spectrin localization to the AIS and its subcellular distribution within neurons, a phenotype that Support Vector Machine classification was able to accurately distinguish from control. Treatment with triciribine also resulted in increased excitability in primary hippocampal neurons. Thus, perturbations to signaling mechanisms within the AKT pathway contribute to changes in βIV spectrin distribution and neuronal firing that may be associated with neuropsychiatric and neurodegenerative disorders.

## Introduction

The axon initial segment (AIS) is a subcellular compartment that exhibits dynamic plasticity of protein interactions which contribute to functional modulation of neuronal excitability (Grubb and Burrone, 2010a; Grubb et al., 2011; Evans et al., 2015). Finely tuned regulation of topology, post-translational modifications and subcellular targeting of AIS proteins is a form of structural plasticity that confers functional specificity to intrinsic firing (Yang et al., 2007; Shavkunov et al., 2012; Hsu et al., 2015) enabling neurons to homeostatically adapt in response to external stimuli. Subtle changes in this regulatory process can increase neuron vulnerability leading to endophenotypes underlying neuropsychiatric disorders and neurodegenerative diseases (Hsu et al., 2014), including bipolar disorder, post-traumatic stress disorder, anxiety, schizophrenia, ataxia, and Alzheimer’s disease in both clinical and pre-clinical animal research (Hsu et al., 2014; Di Re et al., 2017; Huang and Rasband, 2018). Thus, given the demonstrated susceptibility of AIS proteins to disease, understanding the signaling mechanisms that control the AIS protein composition and function is both physiologically and clinically relevant.

The proper distribution and function of voltage-gated Na+ (Nav) channels is critical for the AIS as this compartment acts as the site of the action potential initiation. Crucial to Nav channel clustering to the AIS is a complex nexus of protein:protein interactions (PPI) that incorporates intracellular scaffolding and signaling molecules (Grubb and Burrone, 2010b; Grubb et al., 2011; Jones and Svitkina, 2016; Yamada and Kuba, 2016; Leterrier, 2018) that serve as building blocks anchoring these channels to the local cytoskeleton (Xu and Shrager, 2005; Fréal et al., 2019), an integral step in establishing neuronal polarity and ensuring directional firing (Grubb and Burrone, 2010a, b; Yamada and Kuba, 2016; Huang and Rasband, 2018; Salzer, 2019). Nav channel clustering is dependent upon the initial localization of other scaffolding proteins, especially ankyrin G (Kordeli et al., 1995; Grubb and Burrone, 2010b; Jenkins et al., 2015; Yang et al., 2019) and βIV spectrin (Xu and Shrager, 2005; Yang et al., 2007; Leterrier, 2018; Liu et al., 2020), which are both necessary for the AIS proper formation, and neurofascin, a member of the L1 cell-adhesion molecule family (Boiko et al., 2007; Zonta et al., 2011; Kriebel et al., 2012; Hamdan et al., 2020).

After its initial formation, maintenance of the AIS plays an important role in neuronal plasticity. The size and position of the AIS become highly plastic and responsive to neuronal activity, where an increase in neuronal firing causes a distal shift in the position and a shortening of the AIS (Grubb et al., 2011; Yamada and Kuba, 2016). However, the composition of AIS scaffolding components seems to be relatively stable under normal conditions and altering its composition can cause a loss in neuronal polarity. For example, knocking down ankyrin G after AIS development leads to the acquisition of dendritic features at the former AIS (Hedstrom et al., 2008), while loss of neurofascin disrupts ankyrin G enrichment and relative localization of Nav channels (Zonta et al., 2011; Alpizar et al., 2019).

Posttranslational modifications such as phosphorylation and palmitoylation have been shown to alter protein distribution and membrane targeting of AIS proteins (Garver et al., 1997; Tuvia et al., 1997; Ren and Bennett, 1998; Jenkins and Bennett, 2001; He et al., 2014; Nelson and Jenkins, 2017; König et al., 2018; Salzer, 2019; Yang et al., 2019). Of particular interest for the studies presented here are alterations caused by AKT, GSK3, and Wee1 kinase, as their inhibition was previously shown to either influence interactions between AIS proteins or intrinsic excitability through modulation of Nav channel activity (Shavkunov et al., 2013; Hsu et al., 2015; James et al., 2015; Hsu et al., 2016; Scala et al., 2018). AKT is activated through phosphorylation by PI3K, while GSK3 is a constitutively active kinase whose activity is decreased upon phosphorylation by AKT (Sutherland et al., 1993; Jensen et al., 2007). Disruption of the AKT/GSK3 pathway has been shown to be a risk factor for neuropsychiatric and neurodegenerative disorders (Crofton et al., 2017; Hsu et al., 2017; Scala et al., 2018; Stertz et al., 2020). Wee1 kinase is a complex enzyme that is regulated and degraded by GSK3 through ubiquitination (Watanabe et al., 2004; Li et al., 2012; Penas et al., 2015). Additionally, Wee 1 kinase is a strong inhibitor of cyclin-dependent kinases, which contribute to the repression of Rb protein, and may increase the activity of AKT (Cen et al., 2012), thereby inhibiting GSK3. When dysfunctional, Wee 1 kinase results in loss of neuronal polarity and has been implicated in neurodegenerative changes found in Alzheimer’s disease (Tomashevski et al., 2001; Müller et al., 2010).

Based on this premise, we hypothesized that pharmacological inhibition of AKT, GSK3 and/or Wee1 kinase could have a global effect on AIS protein distribution and neuronal firing. Overall, βIV spectrin was the most sensitive to kinase perturbations, especially the AKT inhibitor triciribine, with effects on its pattern of distribution at the AIS and dendrites. Functionally, perturbations elicited by triciribine were the most robust, leading to increased neuronal excitability. Thus, disruption of βIV spectrin pattern distribution might be a converging node of AKT-dependent signaling pathway mechanisms.

## Materials and Methods

### Cell Preparation

Banker’s style hippocampal neuron cultures were prepared from embryonic day 18 (E18) rat embryos as described in previous work (Shavkunov et al., 2013). Following trituration through a Pasteur pipette, neurons were plated at low density (10^5^ × 10^5^ cells/dish) on poly-L-lysine-coated coverslips in 60 mm culture dishes in MEM supplemented with 10% horse serum. After 24 h, coverslips (containing neurons) were inverted and placed over a glial feeder layer in serum-free MEM with 0.1% ovalbumin and 1 mM pyruvate (N2.1 media; Invitrogen, Carlsbad, CA, United States) separated by approx. 1 mm wax dot spacers. To prevent the overgrowth of the glia, cultures were treated with cytosine arabinoside at day 3 *in vitro* (DIV). Coverslips from independent cultures were grown in separate wells in 24-well plates.

### Cell Treatment

For imaging studies, primary hippocampal neurons (DIV14-DIV18) were exposed to the AKT inhibitor triciribine (25–30 μM; SelleckChem), the GSK3 inhibitor CHIR099021 (5 μM; TOCRIS), the Wee1 kinase inhibitor II (5–10 μM; Sigma-Aldrich) and/or control vehicle 0.5% dimethyl sulfoxide (DMSO) for 12–24 h and processed for immunofluorescence staining (described below). For electrophysiological recordings, primary hippocampal neurons (DIV12-DIV15) were exposed to the AKT inhibitor triciribine (25 μM), the GSK3 inhibitor CHIR099021 (5 μM), the Wee1 kinase inhibitor II (5 μM) and/or control vehicle 0.25% DMSO for 12–24 h before recording (described below).

### Immunocytochemistry

For each protein analyzed, coverslips from at least two independent cultures were grown in separate wells in 24-well plates and considered biological replicates (*n* = 636 cells total, 81–156 analyzed cells per category). Hippocampal neurons (DIV 14) were fixed in fresh 4% paraformaldehyde and 4% sucrose in phosphate-buffered saline (PBS) for 15 min. Following permeabilization with 0.25% Triton X-100 and blocking with 10% BSA for 30 min at 37°C, neurons were incubated overnight at room temperature with the primary antibodies found in Table 1. Neurons were then washed three times in PBS and incubated for 45 min at 37°C with appropriate secondary antibodies. Coverslips were then washed six times with PBS and mounted on glass slides with Prolong Gold anti-fade reagent.

**TABLE 1:** Antibodies used in this study.

Target	Product ID	Type	Source Organism	Dilution	Reagent Provider	RRID
Ankyrin G	N106/36	IgG2a	Balb/C mouse	1:100	UC Davis/NIH NeuroMab, Davis CA, United States	AB_10673030
Ankyrin G	N106/43	IgG1	Balb/C mouse	1:100	UC Davis/NIH NeuroMab, Davis CA, United States	AB_2315803
βIV spectrin	Chicken anti-spectrin	IgY	Chicken	1:5,000	University of Tokyo, Japan	N/A (Gift from Komada Lab)
MAP2	PCK-554P	IgY	Chicken	1:5,000	BioLegend, San Diego CA, United States	AB_291541
MAP2	M3696	IgG	Rabbit	1:100	Sigma-Aldrich, St. Louis MO, United States	AB_1840999
PanNav	ASC-003	IgG	Rabbit	1:200	Alomone Labs, Jerusalem, Israel	AB_2040204
Neurofascin	A12/18	IgG2a	Mouse	1:100	UC Davis/NIH NeuroMab, Davis CA, United States	AB_2877334

### Image Acquisition

Confocal images were acquired with a Zeiss LSM-510 Meta confocal microscope with a 63X oil immersion objective (1.4 NA). Multi-track acquisition was done with excitation lines at 488 nm for Alexa 488, 543 nm for Alexa 568 and 633 nm for Alexa 647. Respective emission filters were band-pass 505–530 nm, band-pass 560–615 nm and low-pass 650 nm. Z-stacks were collected at z-steps of 1 μm with a frame size of 512 × 512, pixel time of 2.51 μs, pixel size 0.28 × 0.28 or 0.39 × 0.39 μm and a four-frame Kallman averaging. Acquisition parameters, including photomultiplier gain and offset, were kept constant throughout each set of experiments.

### Fluorescent Signal Extraction

Acquired Z-stacks were sum projected in FIJI/ImageJ and image-based features were generated from fluorescent intensity profiles of specific analytes, namely PanNav, βIV spectrin, ankyrin G, and neurofascin, measured along the AIS and along representative dendrites of neurons the images. The beginning of the AIS was defined at the start of the MAP2 fading and the entire AIS was traced. AIS that extended past the edge of the image or crossed over another AIS were excluded. For the dendrites, an area outside of the soma was traced for 15 μm. For each selected neuron, fluorescent intensities were computed as follows: (i) A rectangular region-of-interest (ROI) was selected along the AIS and a dendrite in the fluorescent image using a width of 3 or 4 pixels corresponding to a width 1.12 or 1.18 μm; (ii) Background subtraction was performed by selecting an area away from the neurites of each image and subtracting the average value from each point of the ROI. (iii) For the AIS, in order to compare areas of accumulated protein of interest, a method adapted from Guo et al. (2017) was used. Briefly, we created an average of three-point along the AIS and used this average to find the peak of fluorescent intensity for each AIS. From this peak, the point at which the signal decreased below 15% of the peak after background subtraction was used to define the beginning and end of the AIS, creating an analyte specific length from every protein examined. The reported AIS and dendrite intensity values for each protein corresponded to their respective integrated sum (summed pixel intensity/length). The axo:dendritic ratio for each analyte was calculated by dividing the integrated sums of pixel intensity at the AIS by the integrated sum of pixel intensity at the dendrite.

### Electrophysiology

Whole-cell patch-clamp recordings were obtained from cultured mouse hippocampal neurons at 12–15 DIV at room temperature (20–22°C) using a MultiClamp 700B amplifier (Molecular Devices), low-pass filtered at 2.2 kHz, and sampled at 20 kHz using a Digidata 1322A analog-to-digital interface and pClamp9 acquisition software (Molecular Devices). The extracellular bath solution contained (in mM) 140 NaCl, 4 KCl, 2 MgCl_2_, 2 CaCl_2_, 20 HEPES, and 10 glucose, pH 7.4; bicuculline (20 μM), NBQX (20 μM), and APV (100 μM) were added to block synaptic activity mediated by GABA, AMPA, and NMDA receptors, respectively. Recording pipettes (3–4 MΩ) were fabricated from borosilicate glass (WPI) using a two-step vertical puller PC-10 (Narishige), and filled with intracellular solution containing the following (in mM): 120 CH_3_KO_3_S, 10 KCl, 10 HEPES, 10 glucose, 2 MgCl_2_, 0.5 EGTA, 2 MgATP, and 0.5 Na_3_GTP, osmolarity 280–290, pH 7.3, adjusted with KOH. Seal formation and membrane rupture were done in voltage clamp mode at holding potential of −70 mV. After break-in cells were maintained at −70 mV holding potential in voltage clamp mode for ∼1 min and then switched to current clamp mode. To acquire series of trains of evoked action potentials and passive properties all cells then were set to the membrane potential of −60 mV with injection of holding current. Trains of evoked action potentials were induced with a series of square current steps of 500 ms duration and increment of 10 pA. The action potential threshold was defined as the voltage at which the first derivative of the rising phase of the action potential exceeds 10 mV/ms. Passive membrane properties such as input resistance (Rin) were measured with current-clamp recordings from a membrane potential of -60 mV. For determination of Rin the steady-state values of the voltage responses to a series of current steps from -120 to +20 pA with 20 pA increment per step and duration of 200 ms were plotted as a voltage–current relationship. Rin was calculated as the slope of the data points fitted with linear regression.

### Statistical Analysis

For each treatment/protein condition, fluorescent intensity was determined using the profile of the neurite from in Fiji^1^. The integrated sum of fluorescent intensity for each protein were calculated for each AIS and dendrite by dividing the sum of fluorescent intensity by the length of the neurite and then normalized to the corresponding control treatment (DMSO). From these integrated sums, differences in inhibitor treatments were calculated by first checking the distribution of the data using the QQ plots (Supplementary Figure 1). In all cases, the data were log transformed before analysis. Analysis was performed using a Nested One-Way ANOVA in GraphPad Prism version 9.1.0 with cells from individual coverslips considered technical replicates. A Dunnett’s multiple comparisons test was performed in order to compare each inhibitor treatment to DMSO. Cells treated with GSK3 inhibitor and stained for PanNav were cultured and stained separately from AKT and Wee1 kinase inhibitor counterparts and were thus analyzed separately using a Nested *T*-Test to compare them to their own DMSO controls. For electrophysiological recordings, normality was assessed and either a one-way ANOVA or non-parametric Kruskal-Wallis ANOVA was used to determine statistical significance. Results were considered statistically significant if *p* < 0.05.

### Feature Selection

For βIV spectrin, data features for the SVM classifier were calculated as follows: (i) For each neuron (*n* = 150), rectangular regions of interest were manually selected along the first 25 μm of the AIS to control for differences in length. These regions were used to generate fluorescent intensity profiles as a function of the distance from the soma; (ii) Each fluorescent intensity profile was denoised using a low pass filter consisting of a moving window of size 3; (iii) Features were obtained from the fluorescent intensity profile by computing interquantile range, kurtosis, skewness, and root mean square, statistical features selected from the Time Series Feature Extraction Library (Barandas et al., 2020). Finally, the axo:dendritic ratio was concatenated to the above statistical features resulting in a feature vector of length 5 for each neuron.

### Classification

An SVM classifier was trained using the feature vectors computed above. As in standard practice, 70% of the data were randomly selected and used for training the classifier; the remaining 30% were used for testing. Specifically, the data split was 56/24, 47/21, and 50/22 for the Wee1 kinase inhibitor II versus DMSO, GSK3 inhibitor CHIR99021 versus DMSO and AKT inhibitor triciribine versus DMSO experiments, respectively. Each experiment was run 100 times, applying a different, randomly selected 70/30 data split each time, and performance results were averaged. Accuracy was reported as the combined number of true positives and true negatives divided by the total number of classifications.

## Results

**FIGURE 1:**
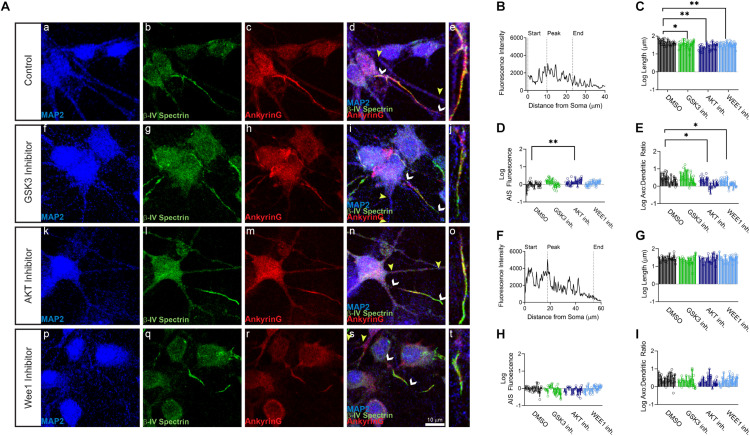
βIV spectrin but not ankyrinG is sensitive to kinase perturbation at the axon initial segment (AIS) and dendrites of primary hippocampal neurons. **(A)** Representative images of expression of (a,f,k,p) MAP2 (blue), (b,g,l,q) βIV spectrin (green), (c,h,m,r) ankyrin G (red), and (d,i,n,s) merge of all channels with white arrows indicating the AIS and yellow arrows indicating a dendrite, (e,j,o,t) zoom to AIS. (a–e) Neurons treated with 0.5% DMSO (control). (f–j) Neurons treated with 5 μM GSK3 inhibitor CHIR99021. (k–o) Neurons treated with 25–30 μM AKT inhibitor triciribine. (p–t) Neurons treated with 5–10 μM Wee1 kinase inhibitor II. **(B)** Profile of βIV spectrin fluorescent intensity from the AIS highlighted in panel (e) with the peak of fluorescent intensity, AIS start and AIS end noted as described in the “Materials and Methods”. **(C)** The length of the βIV spectrin from the AIS is significantly decreased after treatment with the GSK3 inhibitor CHIR99021 (*p* = 0.0203), AKT inhibitor triciribine (*p* < 0.0001), or Wee1 kinase inhibitor II (*p* = 0.0012). **(D)** Fluorescent intensity of βIV spectrin at the AIS is increased following treatment with the AKT inhibitor triciribine (*p* = 0.0091). **(E)** The axo:dendritic ratio of βIV spectrin is decreased in neurons following treatment with either triciribine (*p* = 0.0332) or Wee1 kinase inhibitor II (*p* = 0.0163). **(F)** Profile of ankyrin G fluorescent intensity from the AIS highlighted in panel (e) with the peak of fluorescent intensity, AIS start and AIS end noted as described in the “Materials and Methods”. **(G)** None of the kinase inhibitors examined altered the length of the ankyrin G from the AIS (GSK3 inhibitor *p* = 0.6218; AKT inhibitor *p* = 0.9888; Wee1 kinase inhibitor II *p* = 0.9996), **(H)** the fluorescent intensity of ankyrin G at the AIS (GSK3 inhibitor *p* = 0.2062; AKT inhibitor *p* = 0.3289; Wee1 kinase inhibitor II *p* > 0.9999), or **(I)** axo:dendritic ratio (GSK3 inhibitor *p* = 0.2780; AKT inhibitor *p* = 0.3225; Wee1 kinase inhibitor II *p* = 0.5952). All data are mean ± SEM using a separated scatter graph with bars ^∗^*p* < 0.05 and ^∗∗^*p* < 0.01 by Nested One-Way ANOVA with Dunnett’s multiple comparisons test.

**FIGURE 2:**
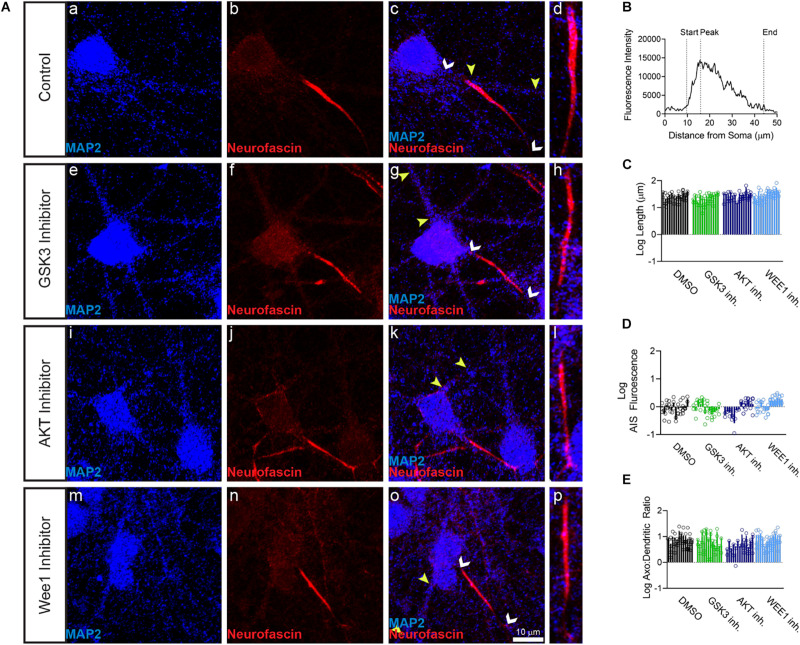
Neurofascin fluorescent intensity is unaffected by kinase inhibitor treatment. **(A)** Representative images of expression of (a,e,i,m) MAP2 (blue), (b,f,j,n) neurofascin (red), (c,g,k,o) merge of all channels with white arrows indicating the AIS and yellow arrows indicating a dendrite, (d,h,i,p) zoom to AIS. (a–d) Neurons treated with 0.5% DMSO (control). (e–h) Neurons treated with 5 μM GSK3 inhibitor CHIR99021. (i–l) Neurons treated with 25–30 μM AKT inhibitor triciribine. (m–p) Neurons treated with 5–10 μM Wee1 kinase inhibitor II. **(B)** Profile of neurofascin fluorescent intensity from the AIS highlighted in panel (d) with the peak of fluorescent intensity, AIS start and AIS end noted as described in the “Materials and Methods”. **(C)** None of the kinase inhibitors examined altered the length of the neurofascin + AIS (GSK3 inhibitor *p* = 0.2426; AKT inhibitor *p* = 0.8066; Wee1 kinase inhibitor II *p* = 0.4303), **(D)** the fluorescent intensity of neurofascin at the AIS (GSK3 inhibitor *p* > 0.9999; AKT inhibitor *p* = 0.9672; Wee1 kinase inhibitor II *p* = 0.1087), or **(E)** axo:dendritic ratio (GSK3 inhibitor *p* = 0.7654; AKT inhibitor *p* = 0.0505; Wee1 kinase inhibitor II *p* = 0.9944). All data are mean ± SEM using a separated scatter graph with bars.

**FIGURE 3:**
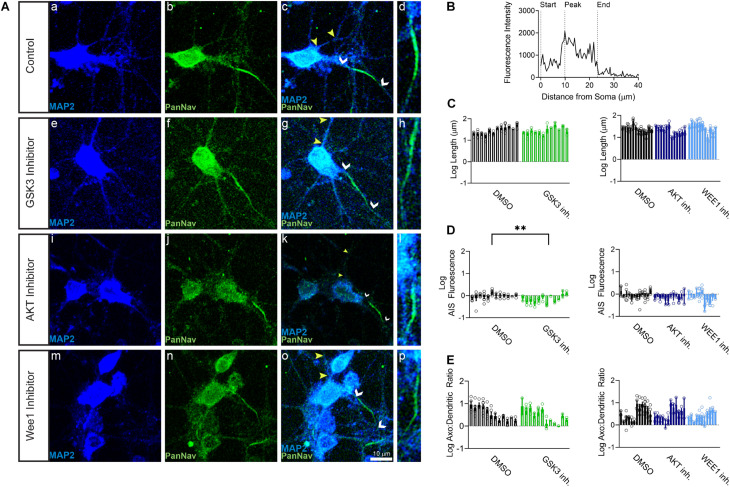
PanNav fluorescent intensity is decreased at the AIS by GSK3 inhibitor treatment. **(A)** Representative images of expression of (a,e,i,m) MAP2 (blue), (b,f,j,n) PanNav (green), (c,g,k,o) merge of all channels with white arrows indicating the AIS and yellow arrows indicating a dendrite, d,h,i,p) zoom to AIS. (a–d) Neurons treated with 0.5% DMSO (control). (e–h) Neurons treated with 5 μM GSK3 inhibitor CHIR99021. (i–l) Neurons treated with 25–30 μM AKT inhibitor triciribine. (m–p) Neurons treated with 5–10 μM Wee1 kinase inhibitor II. **(B)** Profile of PanNav fluorescent intensity from the AIS highlighted in panel (d) with the peak of fluorescent intensity, AIS start and AIS end noted as described in the “Materials and Methods”. **(C)** The length of the PanNav from the AIS is not affected by treatment with the GSK3 inhibitor CHIR99021 (*p* = 0.9610), AKT inhibitor triciribine (*p* = 0.5544), or Wee1 kinase inhibitor II (*p* = 0.5758). **(D)** Fluorescent intensity of PanNav at the AIS is decreased following treatment with the GSK3 inhibitor CHIR99021 (*p* = 0.0061). **(E)** The axo:dendritic ratio of PanNav is not affected following treatment with CHIR99021 (*p* = 0.3904), triciribine (*p* = 0.9767), or Wee1 kinase inhibitor II (*p* = 0.3809). All data are mean ± SEM ^∗∗^*p* < 0.01 by Nested One-Way ANOVA with Dunnett’s multiple comparisons test or Nested *T*-Test using a separated scatter graph with bars.

Primary hippocampal neurons were exposed to the AKT inhibitor triciribine, the GSK3 inhibitor CHIR099021, the Wee1 kinase inhibitor II and/or control vehicle (0.5% DMSO) for 12–24 h and processed for immunofluorescence staining. In control conditions, all AIS proteins were typically enriched at the AIS while being expressed at lower level in the dendrites (Figures 1b,c, 2b, 3b). Treatment with all three inhibitors tested significantly decreased the length of the βIV spectrin positive AIS (Figure 1C). A significant increase was seen in βIV spectrin in the AIS upon treatment with AKT inhibitor triciribine (Figure 1D), while both the AKT inhibitor triciribine and Wee1 kinase inhibitor II increased the immunofluorescence of βIV spectrin in the dendrites (Supplementary Figure 2). The axo:dendritic ratio of the fluorescent intensity of each of the proteins was used as a measure of their subcellular distribution to either the axon or dendrites of the neuron which is a defining characteristic of neurons. This pattern is established during development and is regulated in part by the PI3K/AKT/GSK3 signaling pathway (Arimura and Kaibuchi, 2007; Rolls and Jegla, 2015). In Figure 1E, we show that AKT inhibition resulted in a decrease in the axo:dendritic ratio of βIV spectrin immunofluorescence in neurons treated with either AKT inhibitor triciribine or Wee1 kinase inhibitor II. These changes were driven by an increase in βIV spectrin in the dendrites in the case of Wee1 kinase inhibitor II but were driven by changes in both the AIS and dendrites in the case of AKT inhibitor triciribine. In contrast to this potential sensitivity of βIV spectrin to kinase inhibition, ankyrin G was unaffected by kinase inhibition for any parameter tested (Figures 1F,G). Likewise, neurofascin was also unaffected by kinase inhibition (Figure 2). PanNav immunofluorescence was only affected by GSK3 inhibition, which reduced the intensity of PanNav immunofluorescence at the AIS (Figure 3D), without affecting its axo:dendritic ratio, a result seemingly in contrast with previously published results (Shavkunov et al., 2013), but attributable to differences in the treatment length and concentration of the GSK3 inhibitor.

**FIGURE 4:**
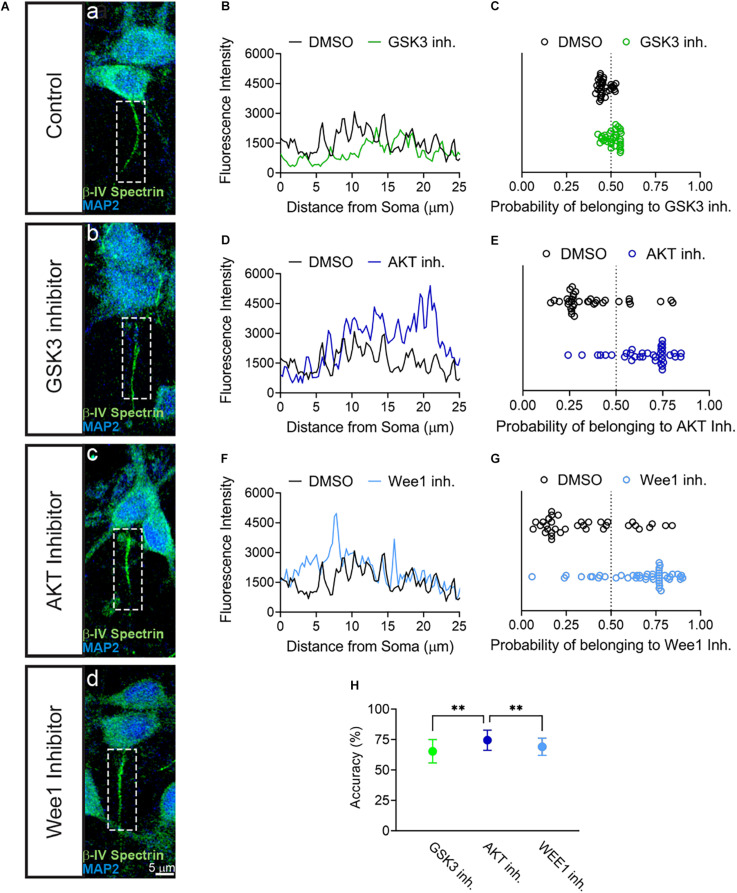
SVM Classification of βIV Spectrin fluorescent intensity accurately sorts AKT inhibitor treated cells from control. (A) Representative images of AIS from neurons treated with (a) 0.5% DMSO (control), (b) 5 μM GSK3 inhibitor CHIR99021 (c) 25–30 μM AKT inhibitor triciribine, (d) 5–10 μM Wee1 kinase inhibitor II with MAP2 in blue and βIV spectrin in green. **(B,D,F)** Profiles of AIS in panel (a–d) showing first 25 μm used for SVM analysis of neurons treated with **(B)** DMSO versus GSK3 inhibitor, **(D)** DMSO versus AKT inhibitor and **(F)** DMSO versus Wee1 kinase inhibitor II. **(C)** Classification of βIV spectrin fluorescent intensity of DMSO versus GSK3 inhibitor treated neurons showing an accuracy of 66% in a sample experiment. **(E)** Classification of βIV spectrin fluorescent intensity of DMSO versus AKT inhibitor treated neurons showing an accuracy of 73% sample experiment. **(G)** Classification of βIV spectrin fluorescent intensity of DMSO versus Wee1 kinase inhibitor II treated neurons showing an accuracy of 70% sample experiment. **(H)** Over 100 tests from the data in panels **(C–E)**, SVM was able to predict cells treated with triciribine or DMSO (74.5% ± 8.3) with a significantly higher accuracy (*p* < 0.0001) compared to CHIR99021 (65.4% ± 9.6) or Wee1 inhibitor II (69.1% ± 7.1). Data are mean ± SD. ^∗∗^*p* < 0.01 by One-Way ANOVA with Dunnett’s multiple comparisons test.

The results above provided evidence for the sensitivity of βIV spectrin to inhibition of AKT. To further establish the sensitivity of βIV spectrin to AKT inhibition, we developed a predictive model based on supervised learning. Specifically, we trained a support vector machine classifier (SVM) using image-based features computed from fluorescent intensity profiles of βIV spectrin measured along axons and dendrites of neurons treated with either DMSO (Figures 4Aa,B,D,F), GSK3 (Figures 4Ab,B), AKT (Figures 4Ac,D), or Wee1 kinase inhibitors (Figures 4Ad,F). SVM prediction accuracies (Figures 4C,E,G) show that the strongest discrimination between treatments for βIV spectrin and control occurs under treatment with the AKT inhibitor triciribine, in which case prediction accuracy is 74.5 ± 8.3%. By comparison, treatment with the GSK3 inhibitor CHIR99021 or Wee1 kinase inhibitor II resulted in an accuracy of 65.5 ± 9.6 and 69.1 ± 7.1%, respectively. Even though the SVM approach indicates sensitivity of βIV spectrin to GSK3, Wee 1 kinase, and AKT inhibitor perturbations, the effect of AKT inhibition is significantly higher than the other two perturbations (Figure 4H). Overall, these converging analyses indicate a remarkable sensitivity of βIV spectrin to inhibition of AKT.

**FIGURE 5:**
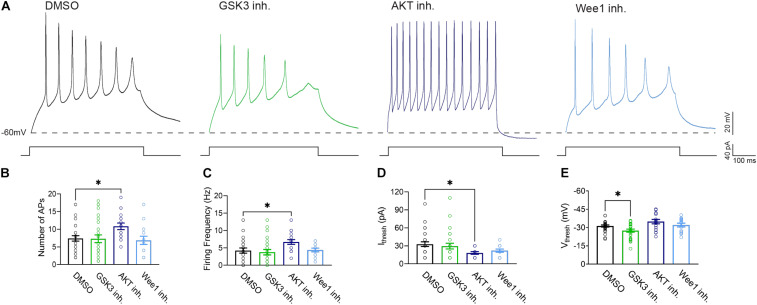
Treatment with AKT inhibitor triciribine increases the intrinsic excitability of primary hippocampal neurons. **(A)** Representative traces of action potential trains from neurons treated with 0.25% DMSO (control), 5 μM GSK3 inhibitor CHIR99021, 25 μM AKT inhibitor triciribine and 5 μM Wee1 kinase inhibitor. **(B)** Treatment with the AKT inhibitor triciribine increased the maximum number of action potentials recorded from primary hippocampal neurons (*p* = 0.0457) compared to DMSO. **(C)** Treatment with AKT inhibitor triciribine increased the firing frequency of primary hippocampal neurons at the current threshold (40 pA) (*p* = 0.0472) compared to DMSO. **(D)** The AKT inhibitor triciribine decreases the current threshold of treated primary hippocampal neurons (*p* = 0.0201) compared to DMSO. **(E)** GSK3 inhibitor CHIR99021 increased the voltage threshold of primary hippocampal neurons (*p* = 0.0475) compared to DMSO. All data are mean ± SEM ^∗^*p* < 0.05 by one-way ANOVA with Dunnett’s multiple comparisons test or Kruskal Wallis ANOVA with Dunn’s multiple comparisons.

To provide functional correlates to confocal image analysis we conducted whole-cell patch-clamp electrophysiology recordings in primary hippocampal neurons to characterize intrinsic firing properties of neurons in response to modulation of the aforementioned kinases using the same inhibitors as described above. To rule out network activity, recordings were performed in the presence of the GABAergic and glutamatergic synaptic blockers bicuculline (20 μM), NBQX (20 μM), and APV (100 μM). In response to prolonged (500 ms) low-amplitude (10–100 pA) depolarizing current injections, neurons treated with triciribine fired repetitively at much higher frequencies, measured as total number of spikes or maximal firing frequency (Figures 5A–C). The mean current (32.86 ± 3.91 pA; *n* = 28) required to evoke action potentials in DMSO-treated control cells (Figure 5D), was significantly larger than in cells treated with triciribine (18.24 ± 2.14 pA; *n* = 17) while the voltage threshold of the first evoked action potential increased upon treatment with CHIR99021 (−27.48 ± 1.2 mV; *n* = 26) (Figure 5E) compared to DMSO control (−31.27 ± 0.84 mV; *n* = 28), an effect which has been previously reported using treatment with 5 μm CHIR99021 (Hsu et al., 2015). The observed changes in maximal firing frequency and action potential current threshold without effects on passive properties and input resistance induced by exposure to triciribine are consistent with increased Nav channel function (Table 2).

**TABLE 2:** Summary of electrophysiology.

	Control	GSK3 inhibitor	AKT inhibitor	Wee1 kinase inhibitor
Voltage threshold (mV)	−31.27 ± 0.84 (*n* = 28)	−27.48 ± 1.26 (*n* = 26)*	−34.88 ± 1.80 (*n* = 17)	−32.07 ± 1.56 (*n* = 14)
Current threshold (pA)	32.86 ± 3.91 (*n* = 28)	29.62 ± 4.72 (*n* = 26)	18.24 ± 2.14* (*n* = 17)	22.14 ± 2.61 (*n* = 14)
Input resistance (MΩ)	626.68 ± 46.49 (*n* = 28)	669.40 ± 46.87 (*n* = 26)	657.49 ± 64.10 (*n* = 12)	791.54 ± 78.36 (*n* = 14)
Tau (ms)	50.75 ± 3.83 (*n* = 28)	57.55 ± 5.23 (*n* = 26)	54.23 ± 5.62 (*n* = 12)	48.05 ± 4.88 (*n* = 14)
Capacitance (pF)	87.20 ± 6.20 (*n* = 28)	95.44 ± 9.84 (*n* = 26)	78.57 ± 12.11 (*n* = 12)	80.09 ± 11.10 (*n* = 14)
Upstroke velocity (dV/dt, mV/ms rise)	56.75 ± 4.02 (*n* = 28)	53.49 ± 5.60 (*n* = 26)	72.75 ± 10.44 (*n* = 12)	58.22 ± 7.47 (*n* = 14)
Downstroke velocity (dV/dt, mV/ms decay)	−25.18 ± 2.08 (*n* = 28)	−22.17 ± 2.55 (*n* = 26)	−23.39 ± 2.65 (*n* = 12)	−22.64 ± 2.85 (*n* = 14)
Latency to first peak (ms)	189.3 ± 23.74 (*n* = 28)	161 ± 23.68 (*n* = 26)	111.85 ± 23.94 (*n* = 12)	162.44 ± 24.31 (*n* = 14)

* indicates p < 0.05 by one-way ANOVA or non-parametric Kruskal-Wallis ANOVA.

Overall, our study reveals a key role of AKT signaling on regulating the molecular composition and function of the AIS, with implications for intrinsic firing of hippocampal neurons that correlate with changes in the subcellular distribution of βIV spectrin.

## Discussion

While a growing body of research suggests that alterations in the AKT/GSK3 pathway activity is a common feature of many neuropsychiatric and neurodegenerative disorders (Emamian et al., 2004; Emamian, 2012; Chen et al., 2015; McGuire et al., 2017; Stertz et al., 2020), studying the consequences of this purported dysfunction is difficult, considering the relatively quick activity of phosphatases *post-mortem* (Li et al., 2005) and the paucity of available brains. In contrast, primary hippocampal neuronal cultures are an excellent *in vitro* model of basic neurobiological functions. Because primary cultured neurons develop fully functional synapses and are readily accessible, these cells are amenable to image-based screenings and electrophysiological studies making them adequate models to screen for the effects of kinase dysregulation in neurons. We focused on the AIS because this subcellular domain is a key regulator of neuronal excitability and plasticity and many AIS proteins have been linked to neuropsychiatric disorders (Hsu et al., 2014; Carroll et al., 2016; Di Re et al., 2017; Sanders et al., 2018; Wang et al., 2018; Hedrich et al., 2019; Ortiz-Gonzalez and Wierenga, 2020; Reynolds et al., 2020). Given the sensitivity of AIS proteins to kinase activity, we determined the effect of kinase inhibition on the subcellular distribution of AIS proteins and found that βIV spectrin was exquisitely sensitive to AKT inhibition. AKT inhibition led to increased fluorescent intensity of βIV spectrin at both the AIS and the dendrites. This finding was corroborated by SVM analysis of βIV spectrin, which was able to discriminate between DMSO and inhibitor treated cells most accurately for AKT inhibition with triciribine. Following all of our image-based analyses, we determined the functional effect of kinase inhibition by using primary hippocampal culture to screen the three kinase inhibitors for effects on neuronal excitability. Based on the sum of these analyses, we determined that inhibition of AKT using triciribine had the greatest effect on neuronal excitability which corresponds with our observed changes in the distribution of βIV spectrin. Though the link between increased βIV spectrin immunofluorescence and increased neuronal firing is not determined here, determining the association between these two phenotypes may lead to a better understanding to neurodegenerative and neuropsychiatric disorders associated with AIS proteins or the AKT pathway.

### Summary of Results

In our image-based analysis of single proteins at the AIS and the dendrites, we determined that βIV spectrin was the most sensitive protein to kinase inhibition, as all kinase inhibitors reduced the length of βIV spectrin staining at the AIS (Figure 1B). In contrast, other proteins were unaffected by kinase inhibition with the exception of PanNav, which was decreased at the AIS of GSK3 inhibitor treated cells (Figure 3D). Notably, among the kinase inhibitors that were tested, triciribine most robustly affected βIV spectrin. In addition to shortening the length of βIV spectrin staining at the AIS, AKT inhibition also increased βIV spectrin fluorescent intensity at both the AIS and dendrites altering its axo:dendritic ratio (Figures 1D,E and Supplementary Figure 2A). The observed change in the βIV spectrin axo:dendritic ratio in response to AKT inhibition was attributable to a greater increase in fluorescent intensity at the dendrites compared to the increase at the AIS suggesting that AKT signaling contributes to βIV spectrin subcellular targeting in the neuron.

To further support our observations from image analysis, we applied a supervised learning approach based on Support Vector Machines (SVM) to assess whether any kinase inhibitors produce measurable alterations on the βIV spectrin distribution at the AIS. Unlike the image analysis we carried out above, which reveals average differences in neuronal populations under different treatments, the SVM approach is more specific. Our SVM analysis addresses the question whether image alterations measured at a single neuron level are reliable predictors of a perturbation, hence establishing a correlation between a perturbation (in this case, induced by a kinase inhibitor) and image-based phenotypes. We trained our SVM model on time-series features from the Time Series Feature Extraction Library (Barandas et al., 2020) which were computed from the axo:dendritic ratio of βIV spectrin and its fluorescence intensity values at the AIS. Such features precisely quantify the pattern expression and fluorescence intensity distribution of βIV spectrin at the AIS. Our SVM approach complements the findings from the primary imaging analysis, namely that βIV spectrin was most sensitive to perturbations induced by inhibition of AKT. Overall, analysis by SVM was able to detect whether a cell was treated with an AKT inhibitor or Wee1 inhibitor with greater than 50% accuracy, but is able to do so significantly more accurately for the AKT inhibitor treated cells (Figure 4H).

We complemented our imaging studies with electrophysiology and found that inhibition of AKT in primary hippocampal neuronal cultures exerts the strongest effect on neuronal excitability as indicated by increased firing frequency and decreased action potential current threshold (Figures 5B–D). The lack of changes in passive properties and input resistance (Table 2) point toward increasing in Nav channel availability (Ali et al., 2018; Scala et al., 2018; Wadsworth et al., 2020) though this does not rule out alterations in other ion channels, which will need further investigation. Understanding the concomitance of the two phenotypes produced upon inhibition of AKT, namely sensitivity of βIV spectrin and increased neuronal excitability, might provide new hypotheses toward understanding AIS plasticity and related neuropsychiatric and neurodegenerative disorders.

Wee1 kinase inhibitor II was included in these studies because of its previously shown effect on the assembly of the Nav1.6 channel and its accessory protein FGF14 (Shavkunov et al., 2013; Hsu et al., 2015). However, this is the first time that the effect of Wee1 kinase inhibition in neuronal cultures has been examined. On the basis of known regulatory links between Wee1 kinase and GSK3 or AKT (Watanabe et al., 2004; Cen et al., 2012; Li et al., 2012; Penas et al., 2015), we expected that inhibition of Wee1 kinase would lead to phenotypes at the AIS that resembled inhibition of either GSK3 or AKT. However, Wee1 inhibition did not resemble entirely either one, but rather resulted in mixed phenotypes. For instance, when analyzing the axo:dendritic ratio of βIV spectrin, Wee1 kinase inhibition led to its increased localization to the dendrites, resembling AKT inhibition. However, Wee1 kinase inhibition did not affect other AIS proteins examined here, nor did it influence neuronal excitability in our cultures. This suggests that there exist multiple distinct mechanisms by which Wee1 kinase differentially affects AIS targets, a hypothesis that warrants future investigations.

## Data Availability Statement

The datasets presented in this study can be found in online repositories. The names of the repository/repositories and accession number(s) can be found below: Mendeley Data repository http://dx.doi.org/10.17632/jgcsykf8xv.1.

## Ethics Statement

The animal study was reviewed and approved by University of Texas Medical Branch Institutional Care and Use Committee.

## Author Contributions

FL, JD, WJH, DL, and CK conceived the experiments. All authors performed the experiments and/or analyzed the data. JD designed the figures and performed statistical analysis. JD and WJH wrote the manuscript with the support of all authors. All authors contributed to the article and approved the submitted version.

## Conflict of Interest

The authors declare that the research was conducted in the absence of any commercial or financial relationships that could be construed as a potential conflict of interest.

## Footnotes

1 https://fiji.sc/
