# Supplementary figures and images for: Inhibition of AKT Signaling Alters βIV Spectrin Distribution at the AIS and Increases Neuronal Excitability
Source: Front Mol Neurosci. 2021 Jun 30;14:643860. doi: 10.3389/fnmol.2021.643860 (PMC8278006; doi:10.3389/fnmol.2021.643860)

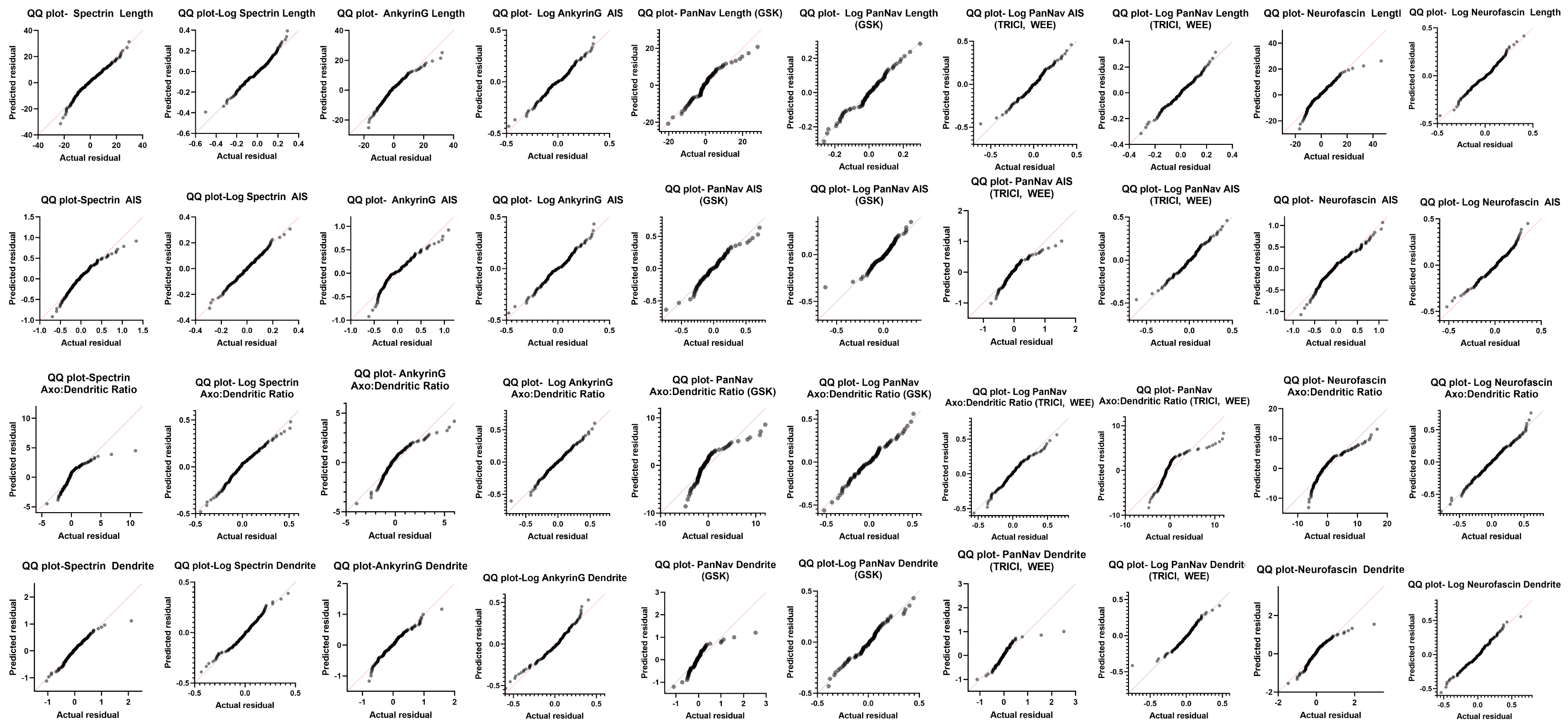

Supplement: Supplementary file 1 [file Image_1.TIF]

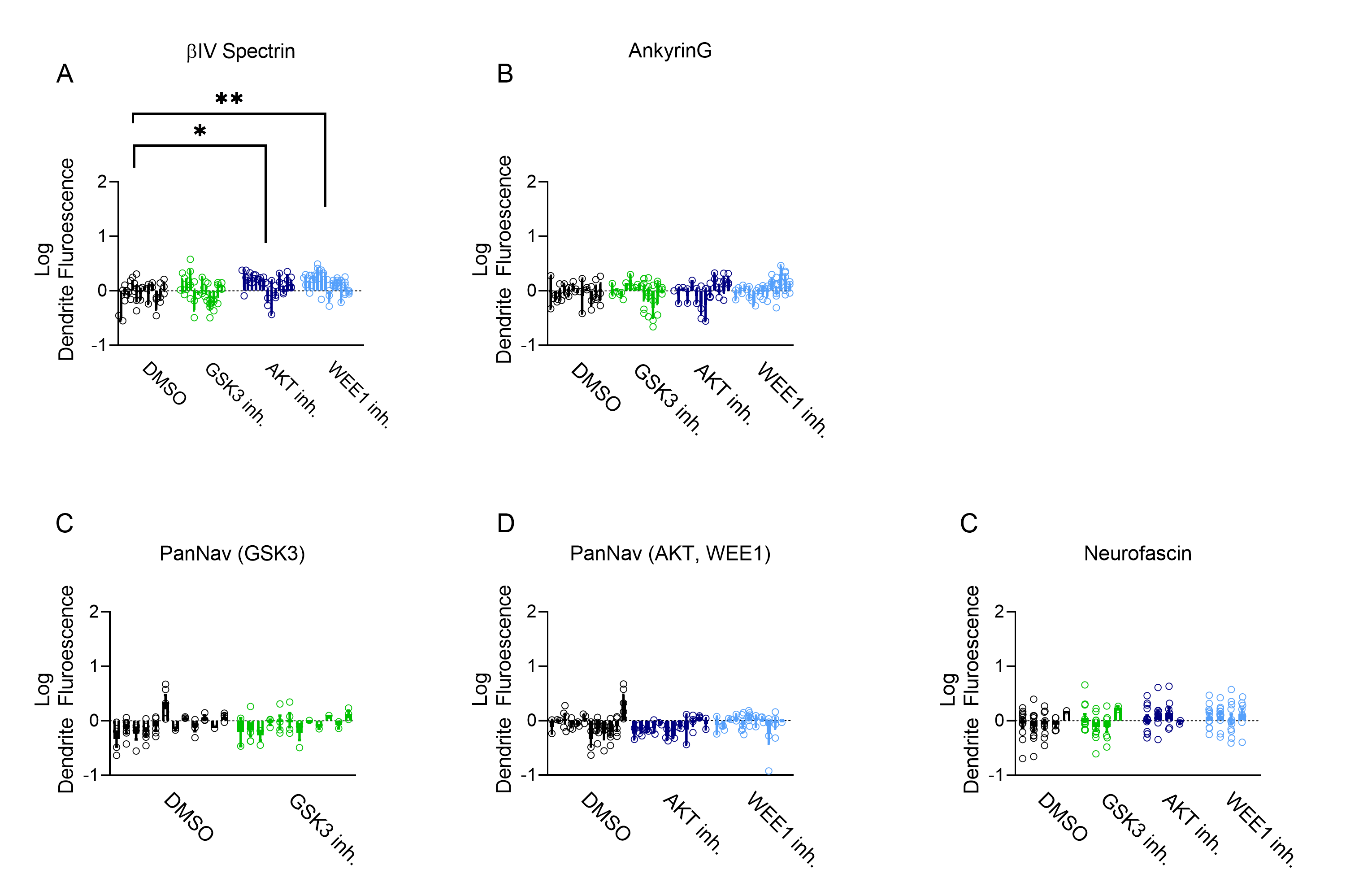

Supplement: Supplementary file 2 [file Image_2.TIF]
